# Conservative Surgery

**Published:** 1881-07

**Authors:** H. I. Ostrom

**Affiliations:** New York


### CONSERVATIVE SURGERY.

H. I. Ostrom, M. D., New York.

Man's mind begins with the concrete, and thence reaches the abstract. At first, his cognitions do not extend beyond a single quality for each object; by this quality the object is recognized. Later, the object is used to the extent which the recognized quality will permit, and from this use, with the purpose of attaining a definite end, and this marks an advanced mental development, arises the power of abstraction.

Evolution of thought, by which all that is of real worth is accomplished, is marked by stages, each of which is recognized as a vantage ground, from which the past may be viewed, and the future, to a certain extent, traced. These cycles of thought assist the great onward movement of humanity, for out of the past the future is born: from the experience of yesterday comes the knowledge of to-day. They are breathing spaces, where the mind matures all that it has acquired, and is able to arrive at a state of absolute perfection, in, at least one branch of conduct, of science, of art, and that perfection is never lost to the world; mankind has gained much, and all future will feel the benefit of the heritage. Let no man think, because his life-work has not been crowned with the success which he hoped to attain, that his efforts have been lost; nothing that is real and earnest is ever lost, for truth must live, and all earnest and faithful work is a part of the truth. Through long years of struggle, through obscurity and persecution, the noble art of surgery has progressed to the honorable position which it now holds among the sciences. Each epoch passed, in attaining the vantage ground upon which we now stand, has been reached by hard-fought battles. All progress is made by men in advance of contemporaneous thought, by men who have worked beyond the period of evolution of their day, and who stand alone, because the reasoning by which they would establish their position is not understood, it is disregarded and thrown aside as the work of a dreamer; but the dreamer has not dreamed—he has thought, and the masses, who cannot follow nor read his thought, call it a vision. Every instrument, every operation of any magnitude, which are now regarded as the glory of surgery, and by means of which thousands of lives have been saved, has been dearly bought; first by persecution and neglect, and then by abuse, for reactionary influence will surely follow extremes, and that which has been discarded, will be lauded to the skies; that which has been held up as the healing serpent, will be dragged into the dust. Only when fanaticism has yielded to reason is it possible to establish truth; not until the mind is open to receive from all sources, and consider without prejudice, or the coloring of any favorite ism, is that state reached which makes the scientist.

The evolutions, through which surgery has passed, have developed its resources, and substituted for harsher methods means which call upon nature and medicine for assistance. That evolution has been in this direction follows from two reasons: *First*, with increased facility and means of action comes, of necessity, conservation of force, and the possibility of attaining greater results with less loss, both of the active and of the passive principle. *Second*, the self-poise and command, which accompany education and thought, furnish the power to await the action of less brutal agents and means, of forces which act more slowly, and hence more surely, and with less commotion of nature, than the uncultered man is willing to employ. This unwillingness arises as much from fear as from ignorance.

What barbarities did the early surgeons practice because of their fear of hæmorrhage? And with what incredulity and puerile reasoning did they oppose the use of the ligature? They knew that the actual cautery would close the bleeding vessel, and they could not understand the application of the ligature, and feared it would not hold; hence, limbs were amputated by the actual cautery, and humanity was burned, and made to suffer untold misery, because of ignorance and prejudice.

Surgery has steadily progressed towards conservatism, has marched onward, leaving behind those who cannot follow, until there has almost come a reaction from the heroic methods, and the knife is brought into unjust disfavor. Rather than open an abscess, the patient is allowed to suffer days and nights of pain; rather than amputate a limb, the part is allowed to slough off, leaving a stump useless to the patient, and an opprobrium to surgery; rather than excise an ovarian tumor, the woman is allowed to die; for, they say, the growth may return. Such practice is not true conservative surgery. It is a mistake to think that the conservative surgeon cannot use the knife; and, if he does, he forfeits his right to be so considered. He will use all other means, if his patient is not losing ground, before he resorts to mutilation; but when these means have failed, none should be more fearless or more ready to operate than the true conservative surgeon.

Conservative surgery taxes the full powers of the man. It is easy to remove a diseased part, but it may be very difficult to cure it. Hence, the conservative surgeon must not only be thoroughly acquainted with his art, but he must possess that nice balancing judgment, which comes only through education and culture. Conservative surgery is the surgery of the future; and the opprobrium of our art will be removed in proportion as the knife becomes less necessary to the saving of life, and men will be true humanitarians when the keen pleasure now felt in an operation gives place to a corresponding satisfaction in a cure effected by less violent means of treatment.

The following cases are selected from those that have recently come under my observation.

### Case 1. Fracture of the Coccyx of Ten Years' Standing, with Severe Coccygeal Neuralgia.

Ten years before I saw the patient, a lady about forty years old, she fell upon her coccyx, causing a fracture of that bone. The fragments united, leaving the bone acutely bent, the concavity directed towards the rectum. From the time of the accident there had been severe pain running from the coccyx, up the spine; at first, paroxysmal, but latterly, almost continuous. The local obstruction and tenderness interfered with defecation, and all the conditions were aggravated during menstruation. One of the principle guides in prescribing for this case, was aggravation upon first moving, or rising from a sitting position, and amelioration upon continued motion; but *Rhus. Puls.* and the other remedies usually associated with these symptoms, administered in various potencies, gave only temporary relief. *Causticum* effected the cure Of course the fractured bone remained in a bent position, but the surrounding muscular structures have adapted themselves to their abnormal relations, and the dependant nervous irritation has been much reduced.

At present, after six months of no treatment, there has not been the slightest return of either pain or soreness. In former days, the coccyx would have been amputated.

### Case II. Fracture of the Fourth Metacarpal Bone.

A young man, to exhibit his strength, struck a hard body with his right fist, and fractured the fourth metacarpal bone. The pain and swelling were considerable, but he sought no treatment until these had abated, two weeks after the accident. Examination showed the fourth metacarpal bone to be fractured in its middle third; between the fragments there was pronounced motion, the upper fragment riding upon the lower. There was an ensheathing callus at the site of the fracture, but this had ceased to assume the vitality of a reparative exudation; the bones moved freely, as in a sheath. As the man's hand was still useful, and he did not feel that he could give any time to the wearing of a splint, or for a radical operation, but must continue at his work, that of a stone-mason, so I consented to try the effect of medicine. He received, night and morning, *Symphytum Off.* In one week the motion between the fragments was reduced, and in one month the bone had firmly united. During this time he worked every day, cutting through cement walls, holding the hammer in his right hand.

The cure must be attributed to the action of the medicine, and not to the irritation produced by the use of the hand; he had continued his work all the two weeks between the fracture and his reporting to me, yet no attempt at uniting was made.

### Case III. Sinuses and Chronic Periostitis of the Femur, of Thirty Years' Standing.

A gentleman between forty-five and fifty years of age, received an injury of right knee when about twenty years old. This was followed by necrosis, for the cure of which the usual operations were performed; the disease, however, was not arrested, as shown by the occasional discharge of sequestra, through the constantly open sinuses above the knee. During these thirty years, the leg has been subject to attacks of erysipelas; the inflammation from this disease and that resulting from the operations caused considerable anchylosis of the knee-joint, which increased with time. My first examination was made in 1878, when I diagnosed slight, if any, necrosis, but serious chronic periostitis about the knee-joint. The counsel of the Old School, however, prevailed, and an operation for the removal of the lower end of the femur was performed by one of our most eminent surgeons.

The operation was a complete failure; not only was there no necrosed bone found, but such an inflammation was set up as to endanger life or seriously suggest amputation of the leg.

Two months after the operation, the condition of the leg was much worse than before, and the surgeon in charge then said that nothing could be done but amputate the leg when the patient's general health was restored. At this stage, I took entire charge of the case, and found the knee bent at an acute angle of 45°, with fine sinuses running in various directions toward the bone; all of which discharged a thin sanious pus; injections showed these sinuses to be connected. There was excessive and continued pain and swelling, with such a degree of sensitiveness, that the clothes could not be borne upon the part. I still could discover no evidence of necrosis; the probe touched the bone in several places, but the surface was smooth.

By the use of injections of *Calendula*, earth poultices, the rubber bandage and the indicated remedy, principally *Silicea*, each in its proper place and until it has accomplished its purpose, this gentleman, in two months, was able to attend to his business; in one year he walked without crutches, and in eighteen months after I began treatment, he could stand and walk better than he had been able to do for years. But one sinus remains, and that discharges a small quantity of healthy pus. Occasionally before a change of weather, especially if the air is cold and dry, he suffers from sciatica; but this is an old trouble, and is caused by some of the nerve filaments passing through old cicatrices, the marks of former operations. This hardened tissue is acted upon by atmospheric conditions and pressure or relaxation alters the nerve circulation.

This rationale is strengthened by the fact that the pain always radiates from one of these spots. It may be said that this case is not yet cured; but practically it is, for all the symptoms of periosteal inflammation are gone, and the probe shows that the single remaining sinus is becoming more and more superficial, it now being impossible to reach the bone.

### Case IV.—Necrosis of the Os Calcis.

The patient, a child of four years, developed necrosis of the os calcis when between two and three years old. When I first saw him, as nearly as the probe could diagnose, one-half of calcis was necrosed. An operation was objected to by the parents; from experiments which I had made with a view to learning the action of *Sulphuric Acid* on diseased and healthy bone, I decided to apply it in this case. There was injected daily into the sinuses, leading to the bone, 10 minims of a fifth solution of strong *Sulphuric Acid*,* care being taken that the syringe should reach the bone before the solution was ejected. The necrosed bone came out, leaving perfectly healthy tissue, which healed in two months from the commencement of the treatment. It is noteworthy that *Sulphuric Acid* used in the strength applied in this case, does not affect the healthy tissues; while it removes the dead bone, it leaves a clean, granulating surface, a more accurate line of demarcation than can be made with the knife. This case is one of several, both in my own practice and that of other surgeons, which have been treated with *Sulphuric Acid* with similar favorable results.

*This use of *Sulphuric Acid* is *not* homœopathic, for it is used in such strength as to destroy tissues. *Sulphuric Acid* is often homœopathic to such conditions, but never is such strength.—Com.

